# Molecular cytogenetic characterization of a de novo derivative chromosome X with an unbalanced t(X;9) translocation in a fetus and literature review

**DOI:** 10.1186/s13039-022-00603-3

**Published:** 2022-06-27

**Authors:** Qiong Wu, Hui Kong, Yanyan Shen, Jing Chen

**Affiliations:** 1 Department of Central Laboratory, Women and Children’s Hospital, School of Medicine, Xiamen University, Xiamen, 361003 Fujian China; 2 Department of Child Health, Women and Children’s Hospital, School of Medicine, Xiamen University, Xiamen, 361003 Fujian China

**Keywords:** Derivative X chromosome, Copy number variation sequencing (CNV-seq), Fluorescence in situ hybridization (FISH), X-chromosome inactivation (XCI)

## Abstract

Partial trisomy 9p is one of the most frequent autosome anomalies in newborn infants featured by craniofacial dysmorphism, intellectual disability and psychomotor growth. Female patients carrying monosomy Xq usually show mild symptoms due to skewed X-chromosome inactivation (XCI). Unbalanced translocation between chromosome X and chromosome 9 is rare in prenatal diagnosis. The skewed inactivation of abnormal X would spread into the extra segment of chromosome 9 presented in the der(X) leading to mild phenotypes. We reported on a fetus with high risk of trisomy 9p(13.32 Mb 9p23-p24.3 duplication)suggested by noninvasive prenatal testing (NIPT), the fetus was normal by ultrasonography. G-banding with trypsin-giemsa (GTG), copy number variations sequencing (CNV-seq) and fluorescence in situ hybridization (FISH) were carried out to delineate the nature of rearrangement. Final karyotype of the fetus was identified as 46,X,der(X)t(X;9)(q27;p23)dn. An unbalanced X-autosome translocation with a deletion of Xqter-q27.2 and a duplication of 9pter-p23 led to mild phenotypes with no obvious alteration by prenatal ultrasonography, or obvious pathological alterations after pregnancy termination.

## Introduction

Partial trisomy 9p featured by craniofacial dysmorphism and intellectual disability and psychomotor growth is the fourth most frequent autosome abnormality in liveborn infants after trisomy 21, 18 and 13 possible due to these chromosomes as well as 9p are relatively gene poor [1, 2]. Patients carrying partial monosomy Xq usually present mild phenotypes due to possibly skewed XCI. Although patients with unbalanced X:autosome translocations are at risk for X-linked disorders and/or partial autosome trisomy, attenuated phenotypes are frequently observed, for the spread of X-chromosome inactivation into autosomal sequences plays a key role in clinical presentation. The difficulty for such prenatal cases lies in the lack of typical signs and symptoms as postnatal cases. Copy number variations (CNVs) were clinically significant in pregnancies without structural sonographic anomalies, especially for those with a high risk of trisomy 9p suggested by noninvasive prenatal testing (NIPT) [3]. Fluorescence in situ hybridization (FISH) was a reliable method to confirm the positive results of noninvasive free DNA detection and could be used to identify real mosaicism and placental mosaicism by detecting uncultured amniocytes [4]. We report an unbalanced X;9 translocations in a 16-week-old female infant with mild features. To the best of our knowledge, this is the first reported fetus with a de novo der(X)t(X;9)(q27;p23). Our findings expand the unknown spectrum of unbalanced X-autosome translocations, and may provide valuable information for prenatal diagnosis and cytogenetic counseling. Moreover, we prompted that NIPT might play a role in the first trimester screening of sub-chromosomal rearrangement.

## Materials and methods

### Ethical approval

This study was performed in accordance with the Declaration of Helsinki, and approved by the Institutional Review Board (IRB) of Xiamen Maternal and Child Health Hospital (Xiamen, China). All participants in this study agreed to donate the remaining samples and data to scientific research, technical innovation and clinical application after the identifiable personal information was removed. All participants provided their informed consent.

### Noninvasive prenatal testing (NIPT)

Experiments and data analysis were performed using the technology platform provided by Beijing Genomics Institution (Beijing Genomics Institution, BGI, CHINA). 5 mL of peripheral blood was collected from the pregnant woman in EDTA containing tubes. The peripheral blood samples were used to extract cell-free fetal DNA (cffDNA) by using of QIAamp DSP DNA Blood Mini Kit (Qiagen, Germany) following the blood protocol. The high-throughput sequencing technology was used to calculate the relative content of cffDNA. The sequence was compared with the human genome reference sequence map. The proportion of each chromosomal was calculated by using Illumina sequencing analysis viewer 19.1 software. If the trisomy risk index Z value ≥ 3, then it indicates high risk of fetal trisomy.

### Cell culture and karyotype analysis

Cell culture and G-banding karyotype analysis were performed in accordance with standard cytogenetic methods. Fetal cells obtained from amniocentesis and peripheral blood lymphocytes from the expecting mother and the expecting father were cultured with a double-line by using standard methodologies, incubated in 37℃ and 5% CO2 incubator for 7 days and 72 h respectively before harvest. The karyotypes were photographed with Zeiss CCD (Carl Zeiss, Germany) and analyzed with AI imaging system (AI, USA). 20 metaphase karyotypes were counted for each case, and 5 karyotypes were analyzed for each sample, all results were interpreted according to an International System for Human Cytogenetic Nomenclature 2020 (ISCN2020).

### Copy number variation sequencing (CNV-seq)

CNV-seq was performed using Low depth whole genome sequencing (Beijing Genomics Institution, BGI, CHINA) according to manufacturer’s instructions. The procedure included genomic DNA extraction from amniocytes using DNA easy blood & tissue Kit (Qiagen, Germany), the sequencing library construction, sequencing (BGI seq500 sequence), Using burrows-Wheeler aligner (version 0.7.7) to compare and analyze the sequence information with human reference genome (GRCh37, UCSC release hg19), judged whether there was a chromosome aneuploid variation and CNV, and searched ISCA, Decipher, Clinvar, DGV databases, and Pubmed to evaluate the pathogenicity of the detected CNV. The threshold of CNVs was set at 100 kb with marker count ≥ 25.

### Fluorescence in situ hybridization (FISH)

Metaphase FISH on cultured amniocytes and the parents’ peripheral blood lymphocytes were performed using the technology platform provided by Be Reative Lab (BRL, CHINA) using subtelomeric FISH probes of chromosome X (Xpter/Xqter) and chromosome 9 (9pter/9qter) (Vysis, USA) respectively according to the manufacturer’s instructions and to reveal the reciprocal translocations. The procedure included hybridization, stringency washes and fluorescence staining using DAPI. All slides were placed under a fluorescence microscope equipped with a CCD camera (Photometrics, USA) (Carl Zeiss, Germany) and analyzed with cytovision system version probe software (Applied imaging, AI, USA). 20 metaphases were analyzed for each probe.

## Results

### Clinical report

A 31-year-old woman, Gravida 1 Para 0 at 16 + 2 weeks of gestation, referred to our hospital together with her 31-year-old husband. Both the husband and the wife were healthy and unrelated. There was no history of exposure to harmful substances like radiation during pregnancy. Doppler ultrasound image for the pregnant woman in our hospital at 16 + 2 weeks of gestation revealed no obvious abnormalities for the fetus except for the unclear display of the fetal spine due to the body position of the fetus.

### Non-invasive prenatal testing

**Fig. 1:**
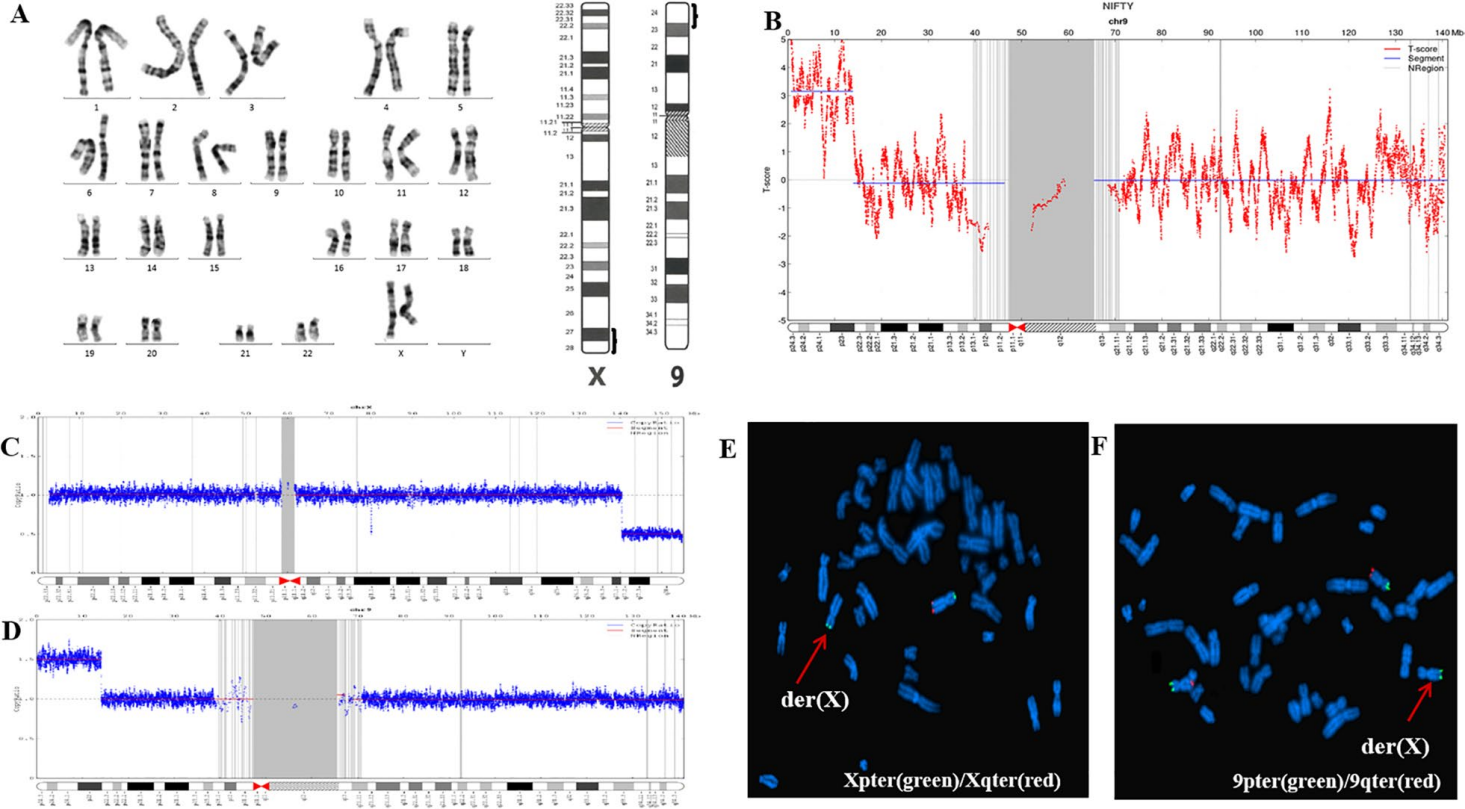
**a** G-banding karyotype of the fetus at 350-band level and idiograms of G-banding patterns for normal human chromosome X and chromosome 9 at 550-band levels. (Left) Karyotype of the fetus revealed no apparent anormality by G-banding. (Right) Idiograms of G-banding patterns showing the rearranged 9p23p24.3 and Xq27.2q28 have similar light and dark bands, brackets indicate the bands rearranged between chromosome X and chromosome 9(cited from ISCN 2020). **b** NIPT result for Chr9. The Chr9 plot shows the mean copy number variation per 20 kb sequencing bin (blue line) versus each 20 kb sequencing bin. The upper dashed line indicates the expected position of the blue line for 100% T9, indicating a 13.32 Mb 9p23-p24.3 duplication staining (609414-13925238). **c**, **d** Molecular karyotype of the foetus showing chromosome X and chromosome 9. **c** Molecular karyotype by CNV-seq revealed the foetus had one copy of seq[GRCH37] Xq27.2q28(140379431-154926263). **d** Molecular karyotype by CNV-seq revealed the foetus had three copies of seq[GRCh37] 9p23p24.3(10001-14098518). **e**, **f** Metaphase FISH on the fetus. **e** Metaphase FISH on cultured amniocytes revealed that the fetus was a carrier of der (X) with two Xpter signals (green) and one Xqter signal (red); arrow indicates the der(X) with no Xqter signal. **f** FISH analysis revealed the fetus had three 9pter signals (green) and two 9qter signals (red) and with one 9pter translocated to the terminal of the non-chromosome 9, which could be inferred as chromosome X by **e**, **f** and DAPI; arrow indicates the der(X)

Maternal free fetal DNA was detected: The NIPT result revealed that the Z score of chromosomes 9 was 3.093; the score suggested that duplication of fetal DNA fragments occur in chromosome 9. There was an approximately 13.32 Mb 9p23-p24.3 duplications (609414–13925238) (Fig. 1b).

### Chromosomal analysis

The conventional cytogenetic analysis revealed no apparent aberrations in the fetus with a karyotype of 46,XX (Fig. 1a). The parents’ karyotypes were 46,XY and 46,XX respectively with no obvious abnormalities (not listed out).

### Copy number variation sequencing

Fetus: seq[GRCH37] del(Xq27.2q28) ChrX: g.140379431-154926263del; seq[GRCH37] dup(9p23p24.3) chr9: g.10001-14098518dup, suggesting a copy number loss of 14.55 Mb in chromosome Xq27.2q28 (Fig. 1c), accompanied by a copy number gain of 14.09 Mb in 9p23p24.3 (Fig. 1d). The expecting father: seq[GRCH37] del(9p13.2p13.2) chr9: g.38265518-38384445 del which had a 118.93 kb copy number loss in chromosome 9p13.2p13.2 (not listed out). The expecting mother: revealed no CNVs (not listed out).

### Fluorescence in situ hybridization

FISH showed that the fetus was a carrier of der(X). Fluorescence signal combined with DAPI analysis revealed that the fetus had two copies of Xpter signals and one copy of Xqter signal, indicating that Xqter was partially deleted. There were three copies of 9pter signals and two copies of 9qter signals indicating a partial trisomy 9pter and one of 9pter was translocated to the end of the long arm of chromosome X (Fig. 1e, f). The fetal mother showed normal Xpqter and 9pqter signals (not listed out). The fetal father revealed normal XYpqter and 9pqter (not listed out).

### Follow up results of fetus

After detailed genetic counseling and full informed consent, though combined with the review of similar patients, a mild phenotypic variation in this fetus might be inferred, the pregnant woman opted to terminate her pregnancy and underwent induced labor in our hospital at 17 weeks of gestation. Pathological examination showed no obvious abnormalities in the induced labor fetus, no abnormality was observed on the face, body weight: 201 g, body length: 20.5 cm, femur length: 2.7 cm. The couple refused pathological dissection on the induced labor fetus.

## Discussion

NIPT was used for the pregnant woman in routine screening of chromosome aneuploidy, and a 13.32 Mb 9p23-p24.3 duplications was suggested prior to ultrasound and invasive prenatal testing. Amniocentesis was performed and a normal karyotype of 46,XX was found for the fetus. The rearranged bands of Xq27.2q28 and 9p23p24.3 are very similar not only in G-dark and G-light bands (Fig. 1a) but also in sizes, 14.55 Mb and 14.09 Mb respectively, which increased the difficulty of identifying chromosome aberrations. This indicated the necessity of combining different cytogenetic/molecular methodologies like CNV-seq and FISH to further exclude unbalanced rearrangements in the fetus and the couple [5]**.** CNV-seq for the fetus revealed a 14.55 Mb copy number loss in Xq27.2q28 and a copy number gain of 14.09 Mb in 9p23p24.3. These results were further confirmed by FISH using probes of 9pter/9qter, Xpter/Xqter to reveal terminal reciprocal translocations.

Fetuses of partial 9p trisomy might be born with a high survival rate for its relatively poor gene region, some might have growth and developmental problems in infancy, including facial deformation, short state, mental retardation, growth retardation [6–9]. Molecular level study showed that 9p22.1-p22.2 was most closely related to the clinical presentation of partial 9p trisomy [10], while the 9p repeat fragment of this fetus covered 9p23p24.3 with no flanking of key areas of trisomy 9p pathogenicity.

Today, the research on X chromosome translocations has focused on skewed X-chromosome inactivation(XCI) and pseudo-autosomal regions (PARs). PARs are classified as PAR1 and PAR2, the latter located on the qter of chromosome X with a size of about 360 kb including the *SPRY3*, *SYBL1*, and *IL9R* genes, which played a key role in antagonistic pathway of fibroblast growth factor (FGF) synthesis, fusion of vesicles and target membrane and occurrence of asthma respectively [11–13]. Mutations or deletions of the foregoing genes would result in haploinsufficiency. In healthy females, one X chromosome undergoes random inactivation, but there are genes that escape X-inactivation, including the genes from the pseudo-autosomal regions (PARs) and some other genes, especially in the Xp region. On the other hand, the Xq region presents fewer genes that escape inactivation, and due to preferential inactivation, deletion of this region may result in fewer phenotypic alterations [14].

X-chromosome inactivation has been localized between Xq13.1 and Xq13.2, with the *XIST* gene playing a key role in chromosome X inactivation. For females carrying unbalanced X**-**autosome translocations, the abnormal X may be tolerated because of the preferential inactivation of the abnormal X chromosome, for the inactivation of the normal X chromosome might result in severe genome imbalance [15–17]. The normal X-chromosome was preferentially inactivated in five patients with balanced X-autosome translocations; while the aberrant X-chromosome was inactivated in most cells from six patients with unbalanced alterations as described by Sisdelli et al. [18]. The spread of X-inactivation into the autosomal regions could affect the phenotype, which could lead to a more severe or a mild clinical presentation depending on the patient's chromosomal constitution. Watanabe et al. reported a 34 years old female with mild phenotypes including congenital heart disease, epicanthal fold, mild intellectual disability, and menstrual irregularity associated with an unbalanced X-autosome translocation, 46,X,der(X)t(X;8)(q28;q13), and suspected that the patient had mild phenotypes due to the inactivation of the abnormal X chromosome 8q13-qter and the presence of a small deletion of Xq28-qter [19].

Patients with unbalanced X:autosome translocations are at risk for X-linked disorders and/or partial autosomal trisomy, however, attenuated phenotypes were frequently observed. It is known that chromosome X inactivation might spread into the autosome part of an unbalanced translocation involving chromosome X and an autosome. A pedigree with 4 women, of 3 different generations, carrying the same cytogenetic anomaly as 46,X,der(X)t(X;7)(q26;q35) with mild clinical presentation mainly characterized by gynecological/hormonal issues and autoimmune disorders was reported to present a skewed X inactivation pattern with a preferential activation of the normal X, in other words, inactivation of the der(X) [20]. Chen et al. described a 33-year-old woman with a karyotype of 46,X,der(X)t(X;5)(q27.3;q32)dn with primary ovarian failure, moderate mental retardation, and mild phenotype of facial dysmorphism [21]. Guo et al. reported a 20 -year-old woman with a karyotype of 46,X,der(X)t(X;1)(q28;q32.1)dn with premature ovarian failure, mental retardation, class I obesity, mild dysmorphism and delayed secondary sexual characteristics [22]. More cases with mild phenotype are as follow: Fusco et al. a woman with 46,X, der(X)t(X;18)(q27;q22)mat with fully attenuated or diminished ovarian reserve (DOR) phenotype in the same family; Sharp et al. a 46,X,der(X)t(X;10)(q26.3;q23.3) with secondary amenorrhea with no abnormal external physical features, all cases were listed as Table 1 [23–26]. Yatsenko et al. reported a case of an X;1 translocation in a 9-month-old female infant with 46,X,der(X),t(X;1)(q28;q32.1)dn with mild dysmorphic features and developmental delay due to late replication of the derivative X in 80% of the observed cells and XCI spread into the translocated 1q segment [27]. Garcia-Heras et al. described girl of a 46,X,der(X)t(X;10)(q26;q21.2) presenting clinical features of distal trisomy 10q, but lacked the classical cardiovascular and renal malformations observed in duplications of 10q24-10qter [28]. These two cases differed from the above cases in that some cells where X inactivation failed to spread into the translocated autosomes, presenting more severe phenotypes. We could conclude from Table.1 that in case of unbalanced translocations between X-autosomes, XCI would happened in the abnormal chromosome X and might spread into autosomes (8 of 11 cases have been verified) presenting mild phenotypes. For this case, the expecting mother refused further testing like,

**Table 1:** Eleven cases of unbalanced X; autosome translocations

Cases	Karyotypes	XCI	Age	Clinical features	References
1	46,X,der(X)t(X;5)(q27.3;q32)dn	Not done	33 y	Primary ovarian failure, moderate mental retardation, and mild phenotype of facial dysmorphism	[21]
2	46,X,der(X)t(X;1)(q28;q32.1) dn	Yes	20 y	Premature ovarian failure, mental retardation, class I obesity, mild dysmorphism and delayed secondary sexual characteristics	[22]
3	46,X, der(X)t(X;15)(q22;q11.2)dn	Yes	3 y	Mild phenotype with normal birth measurements, minor facial dysmorphic features (hypertelorism, short broad nose, and a relatively long philtrum), and moderate developmental delay	[3]
4	46,X, der(X)t(X;18)(q27;q22)mat	Yes	32 y	Fully attenuated or Diminished ovarian reserve (DOR) phenotype in the same family	[24]
5	46,X,der(X)t(X;4)(q22;q24)	Yes	32 y	Secondary amenorrhea, physical exam was within normal limits and was negative for any dysmorphic facial features or physical anomalies	[25]
6	46,X,der(X)t(X;10)(q26.3;q23.3)	Yes	14 y	Secondary amenorrhea with no abnormal external physical features	[6]
7	46,X,der(X)t(X;8)(q28;q13)	Not done	34 y	Mild manifestations, including congenital heart disease, epicanthal fold, mild intellectual disability, and menstrual irregularity	[19]
8	46,X,der(X)t(X;7)(q26;q35)	Yes	fetus 30 y 32 y 60 y	A family with 4 women, of 3 different generations, carrying the same cytogenetic anomaly with mild clinical presentation mainly characterized by gynecological/hormonal issues and autoimmune disorders	[20]
9	46,X,der(X),t(X;1)(q28;q32.1)dn	Yes	11 month	Short stature, unusual head shape, mild dysmorphic features, and mild developmental delay	[27]
10	46,X,der(X)t(X;10)(q26;q21.2)pat	Yes	11 y	Developmental delay and craniofacial, chest, and limb dysmorphism	[28]
11	46,X,der(X)t(X;9)(q27;p23)dn	Not done	fetus	No apparent abnormalities except for the unclear display of the fetal spine	Present study

## Conclusion

In conclusion, we identified a fetus with 46,X,der(X)t(X;9)(q27;p23)dn suggested by NIPT. Combining cytogenetic study, karyotype analysis, FISH and CNV-seq in prenatal diagnosis of unbalanced X-autosome translation was a reliable method [29]. We would like to speculate that the fetus would have mild phenotypes, the possible reason might be that 9p23p24.3 is relatively gene poor and the skewed inactivation of abnormal X would spread into the extra segment of chromosome 9 presented in the der(X) resulting a mild phenotype. To the best of our knowledge, this is the first description of de novo prenatal case with Xq27-qter deletion and 9p23-pter duplication to date. Rare cases might help to understand the genotype–phenotype relationship of such unbalanced X-autosome translocations. In addition, we prompted that NIPT might play a role in the first trimester screening of sub-chromosomal rearrangement.

## Data Availability

All data generated or analyzed in this study are included in this published article. The raw data of CNV-seq and FISH were available upon request.

## Abbreviations

CNVs: Copy number variations
FISH: Fluorescence in situ hybridization
PAR: Pseudo autosomal region
NIPT: Noninvasive prenatal testing
CNV-seq: Copy number variations sequencing
DGV: Database of Genomic Variants
FGF: Fibroblast growth factor
XCI: X chromosome inactivation
Der chromosome: Derivative chromosome
